# How Well Can Centenarians Hear?

**DOI:** 10.1371/journal.pone.0065565

**Published:** 2013-06-05

**Authors:** Zhongping Mao, Lijun Zhao, Lichun Pu, Mingxiao Wang, Qian Zhang, David Z. Z. He

**Affiliations:** 1 Department of Otolaryngology – Head and Neck Surgery, Shaoxing Second Hospital, Shaoxing, P. R. China; 2 Department of Biomedical Sciences, Creighton University School of Medicine, Omaha, Nebraska, United States of America; 3 Ningbo University School of Medicine, Ningbo, P. R. China; University College London, United Kingdom

## Abstract

With advancements in modern medicine and significant improvements in life conditions in the past four decades, the elderly population is rapidly expanding. There is a growing number of those aged 100 years and older. While many changes in the human body occur with physiological aging, as many as 35% to 50% of the population aged 65 to 75 years have presbycusis. Presbycusis is a progressive sensorineural hearing loss that occurs as people get older. There are many studies of the prevalence of age-related hearing loss in the United States, Europe, and Asia. However, no audiological assessment of the population aged 100 years and older has been done. Therefore, it is not clear how well centenarians can hear. We measured middle ear impedance, pure-tone behavioral thresholds, and distortion-product otoacoustic emission from 74 centenarians living in the city of Shaoxing, China, to evaluate their middle and inner ear functions. We show that most centenarian listeners had an “As” type tympanogram, suggesting reduced static compliance of the tympanic membrane. Hearing threshold tests using pure-tone audiometry show that all centenarian subjects had varying degrees of hearing loss. More than 90% suffered from moderate to severe (41 to 80 dB) hearing loss below 2,000 Hz, and profound (>81 dB) hearing loss at 4,000 and 8,000 Hz. Otoacoustic emission, which is generated by the active process of cochlear outer hair cells, was undetectable in the majority of listeners. Our study shows the extent and severity of hearing loss in the centenarian population and represents the first audiological assessment of their middle and inner ear functions.

## Introduction

While many changes in the human body occur with physiological aging, age-related hearing loss ranks among the top three chronic conditions affecting adults over 65 years of age, according to the National Center for Health Statistics [1]. It has been reported that hearing loss affects approximately one-third of adults 61 to 70 years of age and more than 80% of those older than 85 years [1]. Age-related hearing loss, or presbycusis, refers to the physiological age-related changes of the peripheral and central auditory system that lead to hearing impairment and difficulty understanding spoken language. Presbycusis is characterized by decreased hearing sensitivity, reduced speech recognition in a noisy environment, and decreased central processing of acoustic information [2]. The early sign is a loss of hearing sensitivity primarily at high frequencies. Over time, the hearing threshold elevation progresses to lower frequencies. Presbycusis is bilateral, symmetrical, and often sensorineural in origin [2]. Hearing impairment hinders the exchange of information, thus significantly impacting daily life. Loss of hearing in the elderly can also contribute to social isolation and loss of autonomy, and is associated with anxiety, depression, and cognitive decline [2], [3]. There is no known single cause for age-related hearing loss. Most commonly, it is caused by loss of mechanosensitive hair cells in the inner ear as one grows older. However, genetic deficits and repeated exposure to loud noises may play a major role [4]. Smoking and certain medical conditions and medications can aggravate presbycusis [5]–[7].

With advancements in modern medicine and significant improvement of living conditions in the past four decades, the elderly population is rapidly expanding. There are a growing number of populations aged 100 years and older. Although there are many studies documenting the prevalence and degree of hearing loss with advanced age in the United States, Europe, and Asia [8]–[22], auditory function in the population aged 100 years and older has never been evaluated. Therefore, it is not clear how well centenarian listeners can hear. We measured middle ear impedance, hearing threshold, and distortion-product otoacoustic emission (DPOAE) from 74 centenarians living in the city of Shaoxing, China, to evaluate their middle and inner ear functions. This study represents the first audiological assessment of the middle and inner ear functions of the centenarian population.

## Materials and Methods

### Participants

**Figure 1:**
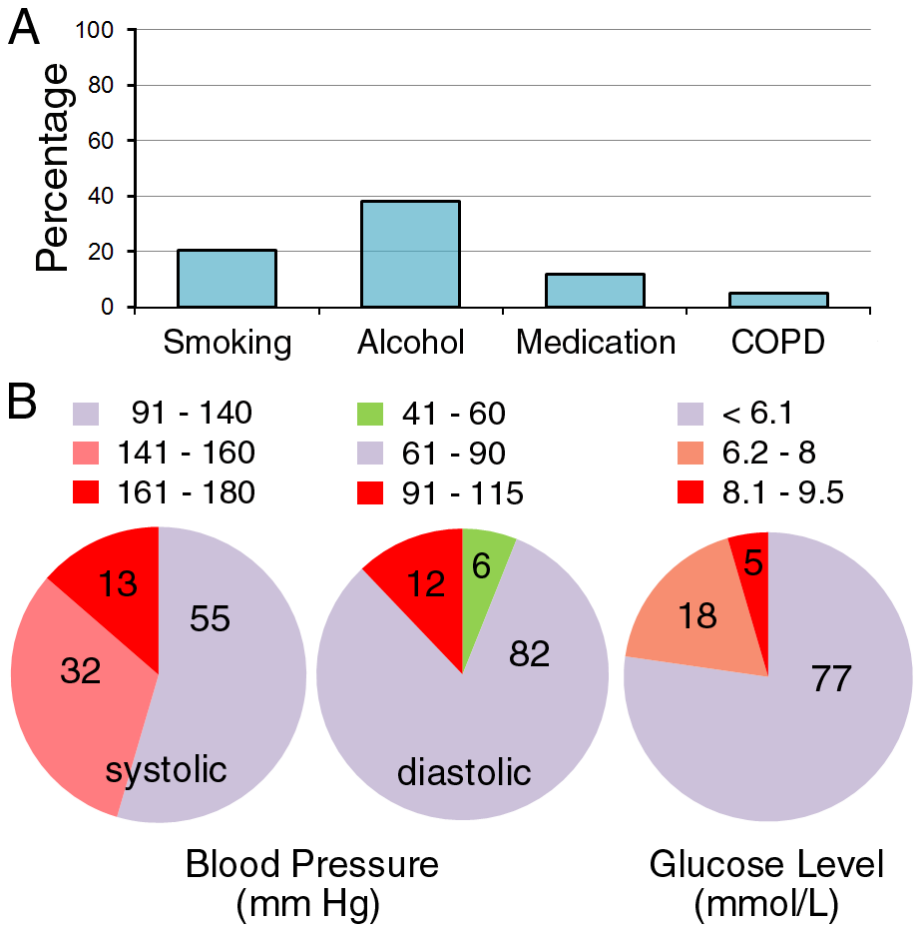
Lifestyle factors and health condition of the centenarian participants. A: Percentage of centenarian subjects who had some of the risk factors for age-related hearing loss. Smoking was defined as consumption of at least half a pack of cigarettes a day for more than a year within the past 10 years. Drinking was defined as consumption of more than 50 ml wine or alcohol on the daily basis for more than a year within the past 10 years. COPD: Chronic obstructive airways disease (diagnosed by a physician). B: Distribution of centenarian subjects (presented as percentage) with different blood pressure and glucose levels. Glucose level presented was based on blood collected 2 to 3 hours after meal. The numbers indicated inside the plots are the percentage.

A total of 74 subjects born before 1911 participated in this study. Hearing tests were part of the physical examinations (including electrocardiogram and laboratory tests of blood and liver function) and mental health evaluation for the centenarians. Their ages varied between 100 and 106 years, with a mean age of 102 years. A questionnaire about ear- and hearing-related medical history, noise exposure (during leisure and work), and self-perceived hearing function was administered as an interview. All participants were farmers with no history of leisure- and/or work-related high intensity noise exposure. Questionnaire data on socioeconomic status, medical history, lifestyle factors, and medication use were obtained as part of the examination. Subjects with a family history of hearing loss and/or a history of ototoxic drug usage were excluded from the study. After excluding subjects with apparent middle ear diseases after otoscopic examination, 68 subjects (21 male and 47 female) were included in the report. The mean age of this group was 102 years. Figure 1 summarizes their lifestyle factors and medical conditions that are known risk factors for aggravating age-related hearing loss. For comparison, we also recruited normal subjects aged between 20 and 25 years and 60 and 65 years. Twenty subjects (equal number of males and females) for each of these two age groups were examined in the same condition as centenarian listeners. The same questionnaire that was used for centenarian subjects was used to obtain medical history and noise exposure history from the younger subjects before hearing tests. Subjects with a family history of hearing loss, history of otologic diseases, and treatment with ototoxic drugs were excluded. Written informed consent was obtained from all participants or their guardians. The procedures described in the present study have been approved by the Institutional Review Board of the Shaoxing Second Hospital.

### Hearing Test Procedures

Pure-tone air-conduction thresholds were obtained in both ears of participants at the frequencies of 250, 500, 1,000, 2,000, 4,000, and 8,000 Hz using a diagnostic audiometer (MADSEN Midimate 622). Bone-conduction thresholds were also obtained at the frequencies of 500, 1,000, 2,000, and 4,000 Hz. No masking was used during test. The audiometer was calibrated in accordance with international (ISO) standards. Testing was completed in a room that met standard requirements. To measure evoked DPOAEs, a MADSEN Capella Cochlear Emissions Analyzer was used. Test frequencies were 500, 1,000, 2,000, 4,000, 6,000, and 8,000 Hz with an f2/f1 ratio of 1.2. The level of f1 was set at 70 dB (maximum output), while the level of f2 was set at 65 dB. DPOAE response was regarded as detectable if the “cubic” distortion tone (DP1) and the “quadratic” distortion tone (DP2) were 6 dB above noise floor. For the assessment of middle ear function, tympanometry was used with a 226 Hz probe tone. An otoscopic examination was performed before all the tests to ensure that the ear canal was clear and that there were no obvious signs of middle ear infection or perforation in the tympanic membrane.

### Statistical Analyses

Middle ear impedance, pure tone thresholds, and DPOAEs were obtained from both ears of each participant. When a participant was unable to hear a tone, 5 dB above the highest audiometer output level was recorded as the threshold. Data were presented as mean and standard deviation (SD) and evaluated with student's t-tests. Statistical significance was assigned to P values of less than 0.01. A p value larger than 0.5 was considered as statistically insignificant. Data presented in this study reflected a sample size of 136, 40, and 40 ears for the centenarian group and the two groups aged 60–65 and 20–25, respectively.

## Results

### 1. Middle Ear Function

**Figure 2:**
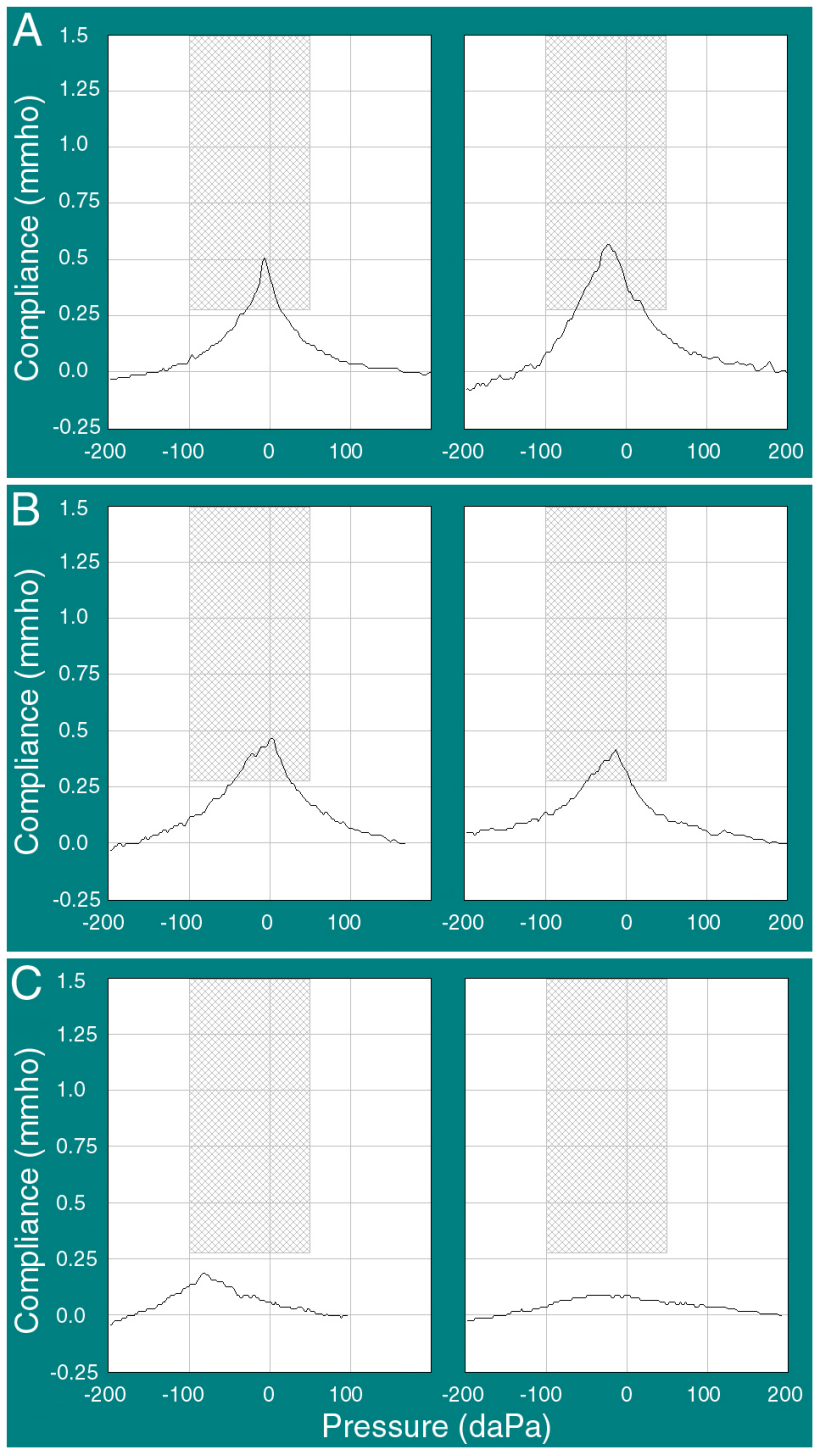
Representative tympanogram obtained from subjects from three different age groups. **A**: 20–25 years old. **B**: 60–65 years old. **C**: ≥100 years old.

**Figure 3:**
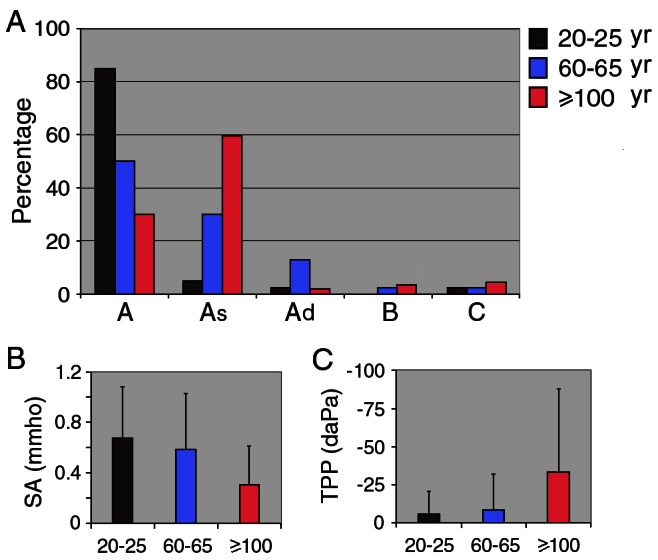
Parameters obtained from tympanometry. **A**: Ratio (presented in percentage) of 5 different types (A, As, Ad, B, C) of tympanogram at different age groups. **B**: Peak compliance (mean ± SD) for the three groups are: 0.31±0.30 (≥100 years old), 0.58±0.44 (60–65 years old), and 0.67±0.41 (20–25 years old) mmho, respectively. Peak compliance of the centenarian group is significantly less than the other two groups. **C**: Middle ear pressure (mean ± SD) for the three groups are: −33.2±54 (≥100 years old), −8.2±24 (60–65 years old), and −5.5±15 (20–25 years old) daPa, respectively.

We used tympanometry to evaluate middle ear function of the centenarian subjects. Tympanometry examines the relationship of air pressure in the external ear canal to impedance of the tympanic membrane and middle ear system. For comparison, middle ear function was also examined from subjects in the age groups of 20–25 and 60–65. The majority of the subjects in the 20- to 25-year-old and 60- to 65-year-old age groups had an “A” type of tympanogram, with compliance peaked near zero decaPascals (Fig. 2A, B). In contrast, most centenarian subjects exhibited an “As” type, with significantly reduced peak compliance (Fig. 2C). We examined the ratio of various types of tympanograms in different age groups and present it in Figure 3A. As shown, only 30% of ears from the centenarian group had the normal “A” type, while the “As” type accounted for ∼60% of the centenarian subjects tested. This is in contrast to more than 80% of ears with “A” type in the group aged 20–25 years and approximately 50% in the group aged 60–65 years. Figure 3B presents the mean and SD values of peak compliance obtained from tympanometry. It is apparent that the peak compliance of centenarian subjects was significantly reduced when compared to those of the younger age groups (p<0.01). The peak compliance between 20–25 and 60–65 year old groups was not statistical different (p = 0.68). Figure 3C shows the mean value of middle ear pressure of the subjects from the three age groups. The middle ear pressure of the centenarian subjects was significantly more negative than the younger subjects (p<0.01). However, the pressure between the two younger age groups was not significantly different (p = 0.51).

### 2. Pure-Tone Behavioral Thresholds

**Figure 4:**
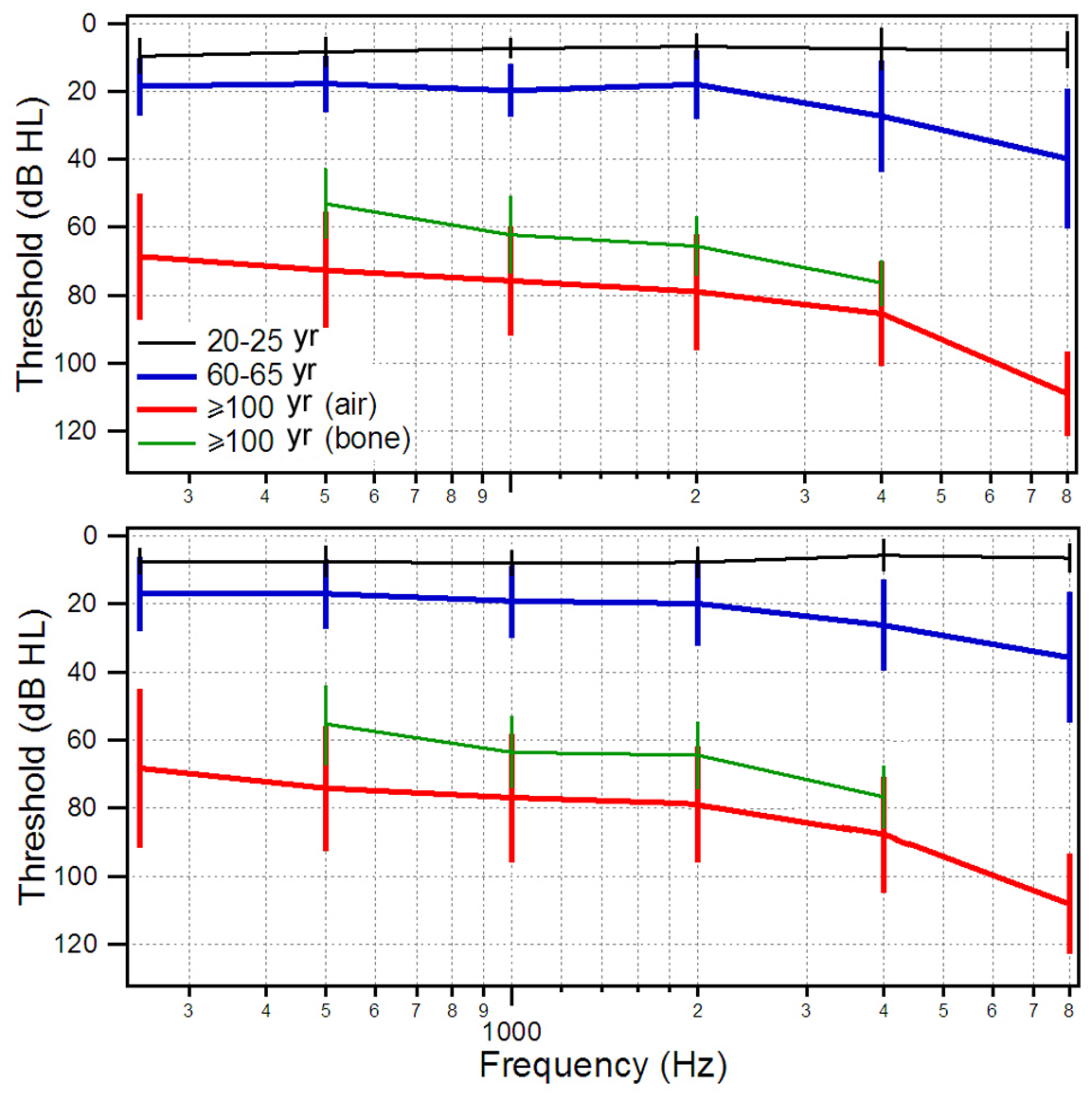
Mean and SD values of pure-tone behavioral thresholds of left and right ears obtained from listeners from different age groups. Bone-conduction thresholds of centenarian subjects are also included. Sixty-eight subjects were included in the centenarians' group, while 20 subjects each participated in the 20- to 25-year-old and 60- to 65-year-old groups.

To determine how well centenarians can hear, we measured air-conduction behavioral thresholds using pure-tone audiometry. For comparison, hearing thresholds of subjects from the two younger groups were also measured. Figure 4 shows the mean and SD values of thresholds of the right and left ears from subjects from the three groups. The mean thresholds of 20- to 25-year-old subjects were all within the normal range (less than 20 dB HL) for the frequency range tested (between 250 and 8,000 Hz). While the mean thresholds of 60- to 65-year-old subjects were within the normal range at low and mid frequencies, their mean thresholds at 4,000 and 8,000 Hz were elevated to 30 and 40 dB HL, respectively. The mean thresholds of centenarian subjects were significantly elevated across all frequencies, with mid and high frequency (4,000 and 8,000 Hz) thresholds exceeding 95 dB HL. Comparison between the audiograms of left and right ears indicated that hearing loss of the centenarian subjects was bilateral and symmetric. We also measured bone-conduction thresholds at the frequencies of 500, 1,000, 2,000 and 4,000 Hz from the centenarian group. As shown in Figure 4, the bone-conduction thresholds were also significantly elevated. However, there was an appropriately 10 to 20 dB difference between the air- and bone-conduction thresholds, with air-conduction thresholds being significantly worse.

**Figure 5:**
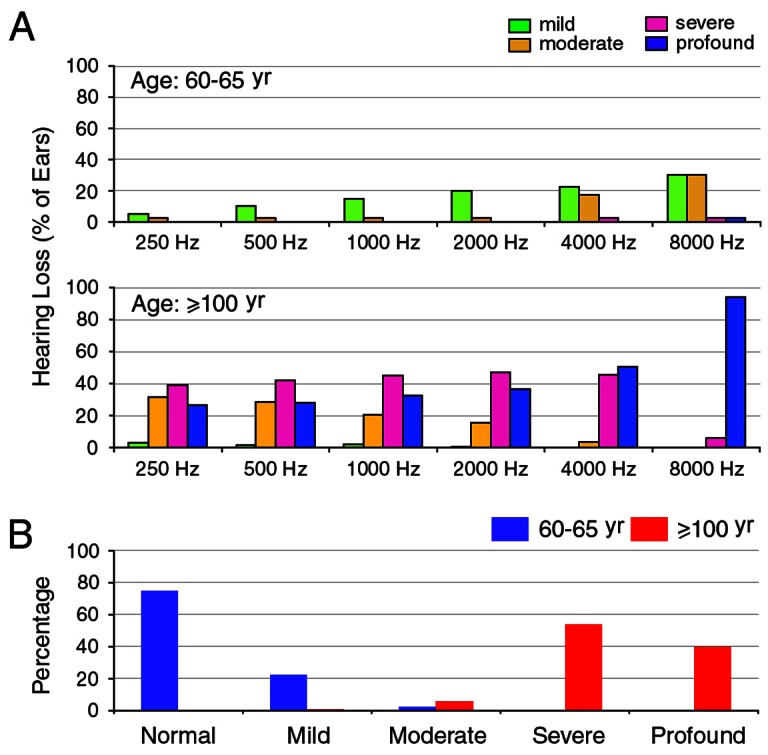
Degree of hearing loss at different frequencies and age groups. **A**: Number of ears (presented in percentage) that had hearing loss at different frequencies from two different age groups. **B**: Percentage of different grades of hearing loss in two different age groups. Hearing loss grade was based on the WHO criterions.

To determine the distribution of different degrees of hearing loss at different frequencies in the elderly population, we graded hearing loss based on the World Health Organization (WHO) criterion [23]. Hearing loss was ranked as mild (26–40 dB HL), moderate (41–60 dB HL), severe (61–80 dB HL), and profound (≥81 dB HL). Figure 5A presents the distribution of ears (as percentage) that had different degrees of hearing loss at various frequencies for centenarians and those aged 60–65 years. As shown, most centenarian subjects had moderate to severe hearing loss at low frequencies. The number of ears that had profound hearing loss increased as frequency increased. At 8,000 Hz, 95% of the centenarian subjects suffered from profound hearing loss. As for the subjects in the 60- to 65-year-old group, the number of ears that had mild to moderate hearing loss also increased at high frequencies. However, only a fraction of subjects in this age group had severe to profound hearing loss (Fig. 5A).

The WHO standards use audiometric threshold values to grade hearing impairment (ranked as mild, moderate, severe, and profound) based on the averages of hearing threshold values at 500, 1,000, 2,000, and 4,000 Hz. Figure 5B presents the percentage of different grades of hearing loss of centenarian subjects, as well as subjects from the 60- to 65-year-old group. As shown, more than 95% of centenarian subjects had severe to profound hearing loss. This is in contrast to 75% of the subjects from the 60- to 65-year old group who showed no sign of hearing loss. 25% of the subjects from the 60- to 65 year old group showed mild to moderate hearing loss.

**Figure 6:**
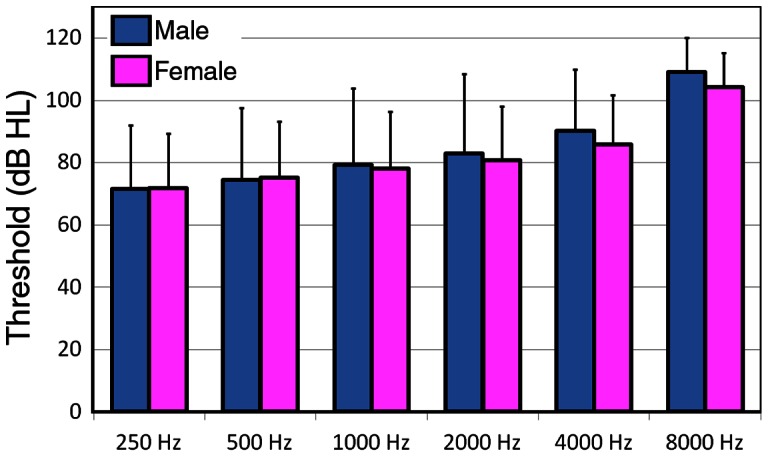
Comparison of hearing thresholds between men and women at different frequencies. Means ± SD are presented. Comparison was made between two genders at each frequencies and no statistical significance in threshold was found at any frequencies (p>0.5).

We also analyzed hearing thresholds of male and female subjects in the centenarian group. Figure 6 presents threshold comparison between male and female subjects at different frequencies. Student's t-test showed that the mean thresholds of male and female subjects at each frequencies were not statistically significant (p>0.5).

### 3. Transient Evoked Distortion Product Otoacoustic Emission

**Figure 7:**
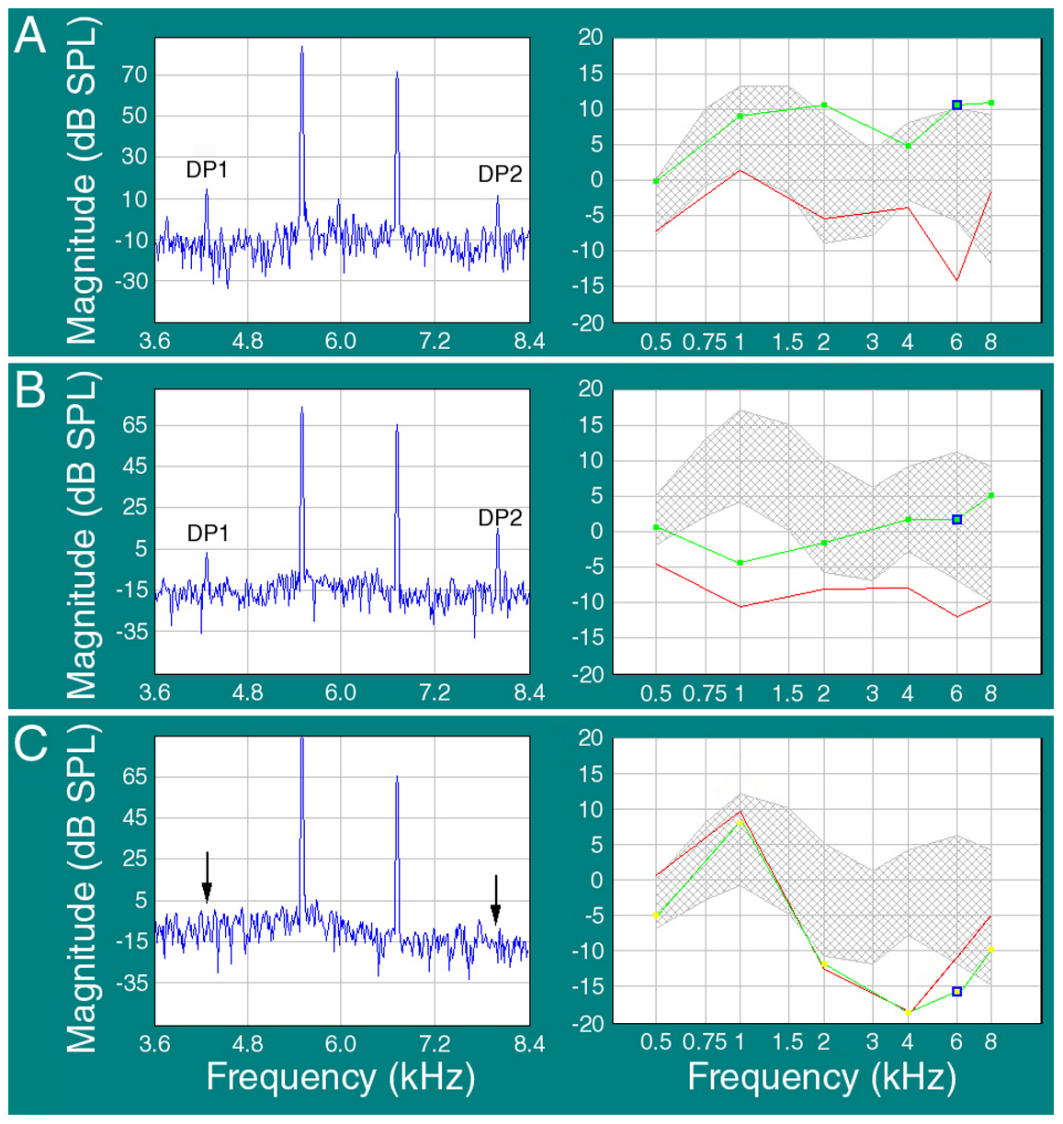
Representative DPOAE responses obtained from listeners from different age groups. **A**: An example of DPOAE response obtained from a subject from the 20- to 25-year-old group. The two probe tones were set at frequencies close to 6,000 Hz. Distortion product one (DP1) and DP2 were clearly visible (∼10 dB above the noise level). The right panel shows the DPOAEs were detected in all test frequencies (500, 1,000, 2,000, 4,000, 6,000, and 8,000 Hz). **B**: An example of DPOAE obtained from a participant from the 60- to 65-year-old group. DPOAEs were also detected in all test frequencies. **C**: Otoacoustic emission from a centenarian subject using the same probe frequencies used in younger groups. DP1 and DP2 were absent (arrows indicate where DP1 and DP2 were expected in the frequency spectrum). The right panel shows that DPOAEs were not detected in all frequencies tested.

A routine non-invasive technique used to evaluate hearing is the otoacoustic emissions test [24]–[27]. We measured DPOAEs from centenarian listeners and two other age groups with test frequencies varying from 500 to 8,000 Hz. Figure 7 shows some examples of DPOAE responses obtained from subjects from different age groups. As shown in the left panels of Figure 7A and B, subjects from younger age groups had robust DPOAEs in response to test tones near 6,000 Hz. DPOAEs are reflected by the presence of DP1 and DP2 in the spectrum analysis. In subjects presented in Figure 7A and B, DPOAEs were present in all frequencies tested (two panels on the right). However, when the same test tones (near 6,000 Hz) were presented to the centenarian subjects, neither DP1 nor DP2 were detected (left panel of Fig. 7C) in majority of the subjects. As shown in the right panel, DPOAEs were not observed in any of the frequencies tested.

**Figure 8:**
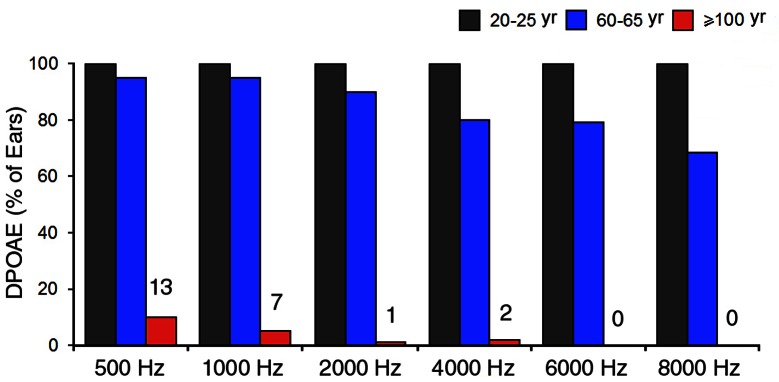
Number of ears (presented in percentage) that had detectable DPOAEs at different frequencies from different age groups. From the centenarian group, 136 ears were measured. The numbers above the red bars indicate the number of ears that had detectable DPOAEs.

To determine how many ears had positive DPOAE responses at different frequencies, Figure 8 exhibits the percentage of ears that had detectable DPOAEs in different age groups. It is apparent that while all subjects from the 20- to 25-year-old group and the majority of participants in the 60- to 65-year-old group had positive DPAOEs at all test frequencies, only a small fraction of centenarian subjects had detectable DPAOEs at low to mid frequencies. None of the centenarian subjects showed positive DPOAEs at frequencies above 6,000 Hz.

## Discussion

The current study is the first audiological assessment of the middle and inner ear functions of centenarian subjects. We show that all centenarian subjects have hearing loss with severity varying from moderate to severe in the low and mid frequencies and from severe to profound in the mid to high frequencies (Figs. 4 and 5). Otoacoustic emissions, which reflect the status of outer hair cells, were undetectable in the majority of the subjects. The elevation of hearing threshold and absence of otoacoustic emissions suggest that sensorineural cause is largely responsible for age-related hearing loss seen in centenarian subjects.

Several large-scale epidemiologic studies were carried out in the United States and Europe to examine the prevalence and degree of hearing loss in the elderly population [9], [11], [12], [15]–[17]. The prevalence of hearing loss shown in these studies varies significantly from study to study. Comparisons of prevalence and degree of hearing loss among different studies are difficult because of the lack of agreement on a standard definition of hearing loss for use in epidemiologic studies, as well as differences in age and sex in the populations tested [16]. Although there are many studies of the prevalence of age-related hearing loss in the United States and Europe, auditory function in the centenarian population has never been examined. The only study that examined hearing from more advanced age was done in an urban Swedish population aged between 85 and 90 [14]. The participants in that longitudinal study were followed audiometrically over a 20-year period from 70 to 90 years of age. The study focused on hearing and its decline during the later time span in which the participants were tested at the age of 85, 88 and 90 years. The results reveal that hearing loss in advanced age progresses only slightly in both men and women. The annual hearing threshold decline is about twice as large in the eighth decade of life as compared with the ninth [14]. Although we did not follow the progression of age-related hearing loss of centenarian subjects in our study, the hearing loss appeared to be much worse than the population aged 85 and 90. We would like to point out that the current study is not an epidemiological study whose goal is to examine prevalence of hearing loss in the general population. Neither was our goal to compare and monitor hearing loss progression over time among different age groups. Our goal is to determine how well centenarian listeners can hear. It is likely that the extent and severity of hearing loss in the general centenarian population are slightly greater than what we reported here. This is because the current study excluded those who already had hearing loss due to genetic deficits (family history), histories of ototoxic drug usage and exposure to impulsive noise, and middle ear diseases. In addition, most subjects in this study were generally in reasonably good health (Fig. 1). It has been reported that changes in the blood supply to the ear because of heart disease, high blood pressure, and other circulatory problems can cause and/or aggravate presbycusis [5], [6].

It has been demonstrated in several well-controlled studies that age-related hearing loss is more prevalent and severe in men than in women, especially at high frequencies [9], [12], [16]. Cruickshanks et al. reported that the mean threshold difference was as large as 20 dB at 4,000 and 8,000 Hz between men and women aged between 60 and 64 years [16]. Such difference is likely due to the fact that men have a greater risk of noise exposure in occupational settings. Interestingly, we did not observe a significant difference of hearing thresholds between men and women at any tested frequencies. The result is not entirely surprising since both men and women in this group already had severe to profound hearing loss. It is conceivable that the difference was minimized when majority of the subjects already suffered from a profound loss of hearing (Fig. 5).

Age-related hearing loss is a natural part of the aging process. Although it is generally accepted that morphological and physiological changes in the middle ear, cochlea, and central auditory system contribute to hearing loss, degeneration of the cochlear hair cells and/or atrophy of stria vascularis play the most important role [5]. A recent study suggests that microRNAs, a class of short non-coding RNAs that regulate the expression of mRNA targets, are important regulators of age-related hearing loss [28]. Animal studies have shown that outer hair cell loss, particularly in the basal turn of the cochlea, is associated with age-related hearing loss [29]. Otoacoustic emissions decrease with age, likely signifying outer hair cell damage [30]. A number of previous studies in humans have shown that as audiometric thresholds become poorer, the magnitude of the DPOAE response decreases and is ultimately eliminated [31]–[39]. Some studies indicate that there is spiral ganglion cell loss during aging [40]. Studies of the human temporal bone from patients with age-related hearing loss have shown a loss of capillaries within the spiral ligament and degeneration of the stria vascularis [2]. It is important to point out that morphological and mechanical changes in the middle ear can also contribute to presbycusis. Such changes can result in reduced function of the tympanic membrane and the acicular chain. We show that the peak compliance of the middle ear was significantly reduced in centenarian subjects (Fig. 3). Furthermore, the bone-conduction thresholds are 10 to 20 dB better than the air-conduction thresholds (Fig. 4), suggesting that conductive hearing loss is partially responsible for age-related hearing loss in centenarian subjects. A number of previous studies have demonstrated the impact of reduced conductive hearing loss on otoacoustic emissions [41]–[46]. Therefore, reduced middle ear function can partially be responsible for the absence/reduction of otoacoustic emissions seen in the centenarian listeners.

Finally, it is worth noting that the audiogram is not a particularly good measure of how one hears in real life. Hearing pure tones under headphones is quite different from listening to complex and dynamically changing sounds coming from different directions. Listening to pure tones in a quiet environment is also different from listening to conversation in background noise. Presbycusis is characterized by decreased hearing sensitivity and reduced speech recognition in a noisy environment. Although we did not perform speech audiometry, many studies have shown that speech discrimination is significantly reduced in the elderly [47]–[49]. The reduced speech recognition is generally believed to be caused by degeneration of the central auditory pathway. Loss of function of the cochlear nerve has been shown in aged animals with reduced synchronous neural activity [50], [51]. This asynchrony may contribute to the decline in temporal resolution during aging. Other animal studies have shown decreased function in the cochlear nucleus [50], [51]. Thus, it is conceivable that morphological and physiological changes in the periphery (middle ear and cochlea) and central auditory system contribute to age-related hearing loss and difficulty understanding spoken language.

In conclusion, we show that although centenarian subjects still retain some residual hearing, more than 95% of them have server to profound hearing loss. It appears that both conductive and sensorineural causes contribute to the age-related hearing loss seen in the centenarian listeners.
